# Correlation between calcium levels and coronary microvascular disease in high-altitude environments: nested case-control study

**DOI:** 10.3389/fcvm.2026.1905691

**Published:** 2026-09-16

**Authors:** Xiyang Rao, Xiao Chen, Wentao Zhang, Juan Lv, Yong Ren, Ruixi Zi, Chunping Bao, Yankun Shi, Lixia Yang, Yu Chen

**Affiliations:** 1 Department of Cardiovascular Medicine, The 920th Hospital of Joint Logistics Support Force of PLA, Kunming, China; 2 Faculty of Life Science and Technology, Kunming University of Science and Technology, Kunming, China

**Keywords:** epidemiology, high-altitude exposure, risk factors, serum calcium, CMVD

## Abstract

**Background:**

Coronary microvascular disease (CMVD) is an emerging concern under high-altitude hypoxia, but data on modifiable correlates are scarce. Serum calcium regulates vascular tone and coagulation, making it a plausible candidate marker. However, its association with CMVD at high altitude has not been systematically examined.

**Methods:**

This nested case-control study enrolled 1,175 participants undergoing high-altitude training at 3,000–3,500 m. Participants were classified into CMVD and non-CMVD groups based on electrocardiogram (ECG) screening and coronary flow reserve (CFR ≤ 2.3) confirmed by transthoracic Doppler echocardiography. Cases (*n* = 235) were individuals with ECG ST-T abnormalities and reduced CFR, while controls (*n* = 940) were randomly matched by age and sex from those without such abnormalities. Pre-training serum calcium (Ca^2+^) levels, along with age, sex, body mass index (BMI), systolic blood pressure (SBP), oxygen saturation (OS), low-density lipoprotein cholesterol (LDL-C), smoking status, and SCOPA-AUT scores, were collected. Multivariable logistic regression was employed to evaluate the independent association between serum calcium and CMVD, adjusting for age, sex, BMI, SBP, and other covariates. Variance inflation factor (VIF < 7) was used to exclude multicollinearity.

**Results:**

Serum calcium levels were significantly higher in the CMVD group than in the non-CMVD group [2.42 (2.32–2.47) vs. 2.37 (2.31–2.42) mmol/L, *P* < 0.001]. Multivariable logistic regression revealed that serum calcium was independently associated with CMVD (OR = 1.39, 95% CI: 1.20–1.61, *P* < 0.001), indicating that each 1 mmol/L increase in calcium was associated with a 39% higher odds of CMVD. Age (OR = 1.05, 95% CI: 1.02–1.09, *P* = 0.001), female sex (OR = 4.13, 95% CI: 1.44–11.89, *P* = 0.009), and SBP (OR = 1.02, 95% CI: 1.00–1.03, *P* = 0.009) were also independently associated with CMVD. Subgroup analyses showed consistent associations across most strata, except in underweight and obese BMI categories and former smokers.

**Conclusion:**

In this nested case-control sample of high-altitude trainees, higher serum calcium levels were significantly associated with an increased prevalence of CMVD. These findings suggest that serum calcium may serve as a potential screening indicator for CMVD in high-altitude settings.

## 1 Introduction

Coronary microvascular disease (CMVD) refers to structural or functional abnormalities of the small arteries and arterioles that supply the myocardium (1). Although coronary microvessels account for approximately 75% of total coronary resistance and their dysfunction directly affects myocardial perfusion (2), CMVD is often underdiagnosed due to phenotypic heterogeneity and limited routine diagnostic tools. Clinically, it may present as atypical chest pain or myocardial ischemia even in the absence of significant large-vessel stenosis, and it substantially increases the risk of adverse cardiovascular events.

With advancements in functional assessment techniques, diagnostic tools for CMVD include invasive methods such as vascular reactivity tests, coronary flow reserve, microvascular resistance reserve, and continuous thermodilution (3–7), as well as non-invasive approaches including positron emission tomography and transthoracic Doppler echocardiography (8). Among these, transthoracic Doppler echocardiography-derived coronary flow reserve (TDE-CFR) has become a clinically accessible, repeatable, and non-invasive tool that can identify individuals with impaired microcirculatory function within the spectrum of non-obstructive coronary artery disease.

High-altitude environments, characterized by low atmospheric pressure and hypoxia, trigger multi-level cardiovascular adaptations, including sympathetic activation, increased heart rate and blood pressure, and compensatory coronary flow changes. Wyss et al. demonstrated in an acute hypoxia experiment simulating 4,500 m that while hypoxic exposure may increase coronary blood flow to maintain oxygen supply, microcirculatory reserve may still be limited, potentially unmasking underlying microvascular vulnerability (9). This suggests that high-altitude exposure could amplify CMVD-related ischemic phenotypes and create additional risk stratification needs in this specific population.

Calcium plays a pivotal role in vascular smooth muscle contraction–relaxation coupling, endothelial function, and coagulation. Experimental and observational data indicate that elevated serum calcium is associated with increased cardiovascular events, shortened clotting time, and enhanced thrombus strength (10). In the setting of hypoxia and sympathetic overactivity, even subtle disturbances in calcium homeostasis may be amplified, potentially affecting microcirculatory resistance and endothelial reactivity.

Despite the biological plausibility linking calcium to microvascular dysfunction, population-based evidence on the association between baseline serum calcium levels and CMVD in high-altitude settings remains scarce. Previous studies have predominantly focused on sea-level populations or have not validated outcomes using objective microcirculatory functional metrics. To address this gap, the present study aims to evaluate whether pre-altitude serum calcium levels are associated with CMVD prevalence in individuals undergoing high-altitude training at 3,000–3,500 m, while accounting for potential confounders including age, sex, body mass index, and systolic blood pressure.

## 2 Methods

### 2.1 Research design

This study employed a nested case-control design. A cohort of 4,000 participants from the 920th Joint Logistics Support Force (JLSF) who underwent pre-altitude health examinations between January and June 2022 was prospectively enrolled. All participants then underwent 3–6 months of continuous altitude training at 3,000–3,500 m. After the training period, individuals exhibiting ST-T wave abnormalities on electrocardiograms were identified as potential cases. These individuals then underwent further evaluation by transthoracic Doppler echocardiography; those with a coronary flow reserve (CFR) ≤ 2.3 were confirmed as CMVD cases. For each confirmed case, four controls without ECG abnormalities were randomly matched by age (±2 years) and sex, yielding a final analytical sample of 1,175 participants (235 cases and 940 controls).

In this study, “high-altitude environment” is defined as the high-altitude training area located in Yunnan Province, China, at an elevation of 3,000–3,500 m above sea level. This region is characterized by low atmospheric pressure (approximately 65%–70% of sea-level pressure), chronic hypobaric hypoxia (oxygen partial pressure approximately 14–15 kPa), low annual average temperature (approximately 5–10 °C), low humidity, and strong ultraviolet radiation. All participants underwent continuous altitude training at this elevation for 3–6 months prior to outcome assessment.

### 2.2 Inclusion and exclusion criteria

Participants with pneumonia, asthma, established clinical diagnosis of hypertension, pathological Q waves, arrhythmias, a history of myocardial hypertrophy, or pre-altitude ECG abnormalities (including ST-T alterations) were excluded from the study. Additionally, individuals who had not undergone high-altitude training were also excluded. To verify eligibility, electronic medical records from the preceding six months were reviewed to exclude participants who had suffered from acute mountain sickness, significant training-related injuries, or COVID-19 infection. Individuals with post-altitude ECG ST-T abnormalities were selected from the overall population as potential cases, and four controls without ST-T abnormalities were randomly matched by age and sex, forming the final sample (Figure 1).

**Figure 1:**
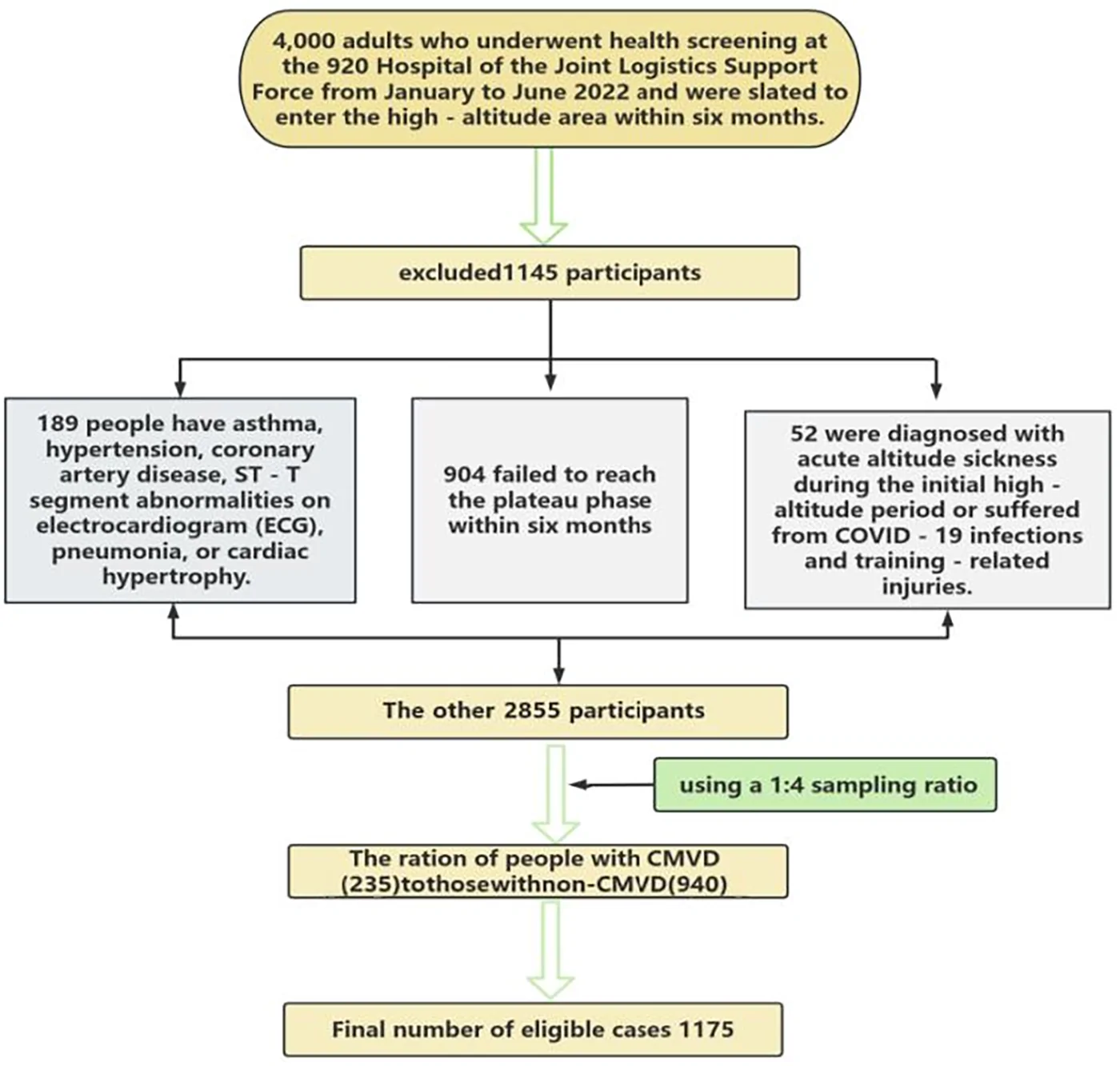
The procedure for screening.

### 2.3 Variables and measurements

The study used clinical data from health examinations conducted within 1 month prior to altitude deployment and immediately after completion of the 6-month altitude training period as initial variables. The study incorporated fundamental details such as age, sex, and smoking status, along with measurements of body mass index (BMI), systolic blood pressure (SBP), pre-altitude low-density lipoprotein (LDL) levels, and SCOPA-AUT (Scales for Outcomes in Parkinson's Disease–Autonomic Dysfunction) scores, a standardized questionnaire assessing autonomic symptoms. Covariates were selected based on literature-supported hypotheses (11). The SCOPA-AUT score was assessed using standardized scales (12). All hematological tests were conducted at the Joint Logistics Support Force 920th Hospital's health examination center.

### 2.4 Coronary microvascular disease

Participants underwent a two-step diagnostic process for CMVD.

Step 1 – Electrocardiogram (ECG) screening: Participants refrained from any physical exertion for a minimum of 48 h prior to ECG testing to minimize exercise-related influences and electrolyte disturbances on ST-T segment measurements. Myocardial ischemia was identified based on the following ECG criteria: (1) J-segment or ST-segment depression of at least 0.5 mm or downward-sloping ST segments in at least two adjacent leads; (2) T-wave inversion of at least 1 mm in at least two consecutive leads predominantly exhibiting R waves (R/S ratio >1) (13).
Step 2 – Coronary flow reserve (CFR) confirmation: For participants with ECG ST-T abnormalities, portable echocardiography was first used to exclude myocardial disease or pulmonary hypertension. Subsequently, transthoracic Doppler echocardiography (TDE) was employed to assess CFR. A high-frequency probe (5–7 MHz) was used to scan the middle and distal left anterior descending artery (LAD) in the anterior chest region. Microcirculatory vasodilation was induced by intravenous adenosine infusion at 0.14 mg/kg/min. Tissue Doppler imaging measured diastolic blood flow components, and integrated ultrasound system software was used for pulse Doppler signal analysis to assess blood flow velocity, with data processed offline (14, 15). CFR was calculated as the ratio of hyperemic to baseline average flow velocity. A CFR ≤ 2.3 was defined as impaired coronary microcirculation function (16).

### 2.5 Bias

To reduce selection bias, we employed a matched sampling strategy (1:4 matching by age and sex) to select controls from the same source population. To reduce information bias, standardized measurement tools were applied. To reduce confounding bias, the variance inflation factor (VIF) for each risk factor in the model was calculated, and factors with VIF ≥ 7 were excluded due to multicollinearity (17). A dual verification system was established to evaluate the results of electrocardiograms (ECGs), overseen by a senior cardiologist possessing more than eight years of expertise. Additionally, transthoracic Doppler echocardiography (TDE) was employed to assess coronary flow reserve (CFR) in individuals exhibiting ST-T abnormalities in their ECG readings. While invasive coronary angiography is regarded as the definitive method for diagnosing CMVD through the evaluation of CFR and the index of microcirculatory resistance (IMR), the present study did not extensively utilize TDE screening or invasive coronary angiography. This limitation has led to certain inconsistencies when compared to the established gold standard.

### 2.6 Sample size

This research conducted a *post-hoc* power analysis to evaluate the statistical efficacy in identifying particular effect sizes based on the existing sample size (18). The analysis utilized independent/paired *t*-tests and proportion comparisons, with a significance level (*α*) set at 0.05 (two-tailed). Employing the observed effect size Cohen's *d* = 0.5, a group difference of 5%, and a total sample size *N* = 1,175, calculations were conducted via G Power software. The results indicated that the statistical power exceeded 99.9%, significantly higher than the academic standard benchmark of 80%. Even when the minimum detectable effect size (Cohen's *d* = 0.2) was selected, the power reached 85%, suggesting that the experimental design has sufficient sensitivity to detect the observed magnitude of the effect.

### 2.7 Statistical methods

Continuous variables exhibiting a normal distribution were described using mean and standard deviation, while those that deviated from normality were represented by median and interquartile range. Statistical significance was assessed using *t*-tests, rank-sum tests, or chi-square tests, with *P*-values calculated accordingly. Baseline characteristics were compared between the CMVD and non-CMVD groups; variables with *P* < 0.05 in this baseline comparison were identified as potential candidates. Univariate logistic regression analysis was then conducted on these candidate variables, and those with *P* < 0.05 (age, sex, SBP, and serum calcium) were entered into the initial multivariable logistic regression model. A backward stepwise selection procedure based on the likelihood ratio test was then applied (removal criterion *P* ≥ 0.05); the four entered variables all remained statistically significant and were retained in the final model. Adjusted odds ratios (OR) and 95% confidence intervals (CI) were calculated for the final model. All statistical analyses were conducted using R (4.2.3) software.

### 2.8 Ethics approval and consent to participate

In alignment with the ethical standards set forth by the Helsinki Declaration, the experimental protocol was formulated and received approval from the Human Ethics Committee at the 920th Hospital of the Joint Logistics Support Force (Reference number: 2022-135-01). Furthermore, written informed consent was acquired from either the participants themselves or their guardians.

## 3 Results

### 3.1 Data description

The present study encompassed 4,000 adults who participated in health assessments at the 920 Hospital of the Joint Logistics Support Force between January and June 2022, with plans to enter a high-altitude region within the subsequent six months.

During the research process, 1,145 participants were excluded owing to various conditions such as asthma, hypertension and coronary artery disease, ST-T segment abnormalities on electrocardiogram (ECG), pneumonia, or cardiac hypertrophy. Among the remaining participants, 904 failed to reach the plateau phase within six months, while 52 were diagnosed with acute altitude sickness during the initial high-altitude period or suffered from COVID-19 infections and training-related injuries. Ultimately, 2,855 valid samples were collected.

The study included 1,175 participants, with 235 having CMVD and 940 without. The study comprised 16 female participants and 1,159 male participants. The findings indicated that age, gender, SBP, OS, and Ca^2+^ were statistically significant independent factors contributing to an elevated risk of CMVD across the two demographic groups. The analysis conducted between the two cohorts indicated that factors such as age, sex, SBP, OS, and Ca^2+^ were independently linked to a heightened risk of CMVD. Specifically, The levels of Ca^2+^ were elevated in the CMVD group: 2.42 [2.32;2.47] vs. 2.37 [2.31;2.42] (*P* < 0.001). The percentage of female participants in the CMVD group was notably greater than that observed in the non-CMVD group (*P* = 0.007).

The median age was similar between the groups: 24 [22;29] for CMVD and 24 [22;26] for non-CMVD (*P* = 0.001). SBP was marginally elevated in the CMVD group: 119 [110;125] vs. 117 [110;120] (*P* = 0.004). OS was comparable between the groups: 93 [92;96] for CMVD and 93 [92;95] for non-CMVD (*P* = 0.038). The CMVD group exhibited a marginally elevated BMI: 22.59 [20.94;24.38] vs. 22.15 [20.98;23.62], but the difference was not statistically significant (*P* = 0.07). LDL-C, smoking status, and SCOPA-AUT scores showed no significant differences between the groups (*P* > 0.05) (Table 1).

**Table 1:** Baseline characteristics of CMVD cases and control group.

Characteristics	Non-CMVD (*n* = 940)	CMVD (*n* = 235)	*P* value
Age/years, [IQR]	24 [22; 26]	24 [22; 29]	0.001
Sex			0.007
Woman	8 (0.9%)	8 (3.4%)	
Man	932 (99.1%)	227 (96.6%)	
BMI/kg/m^2^, [IQR]	22.15 [20.98; 23.62]	22.59 [20.94; 24.38]	0.070
SBP/mmHg, [IQR]	117 [110; 120]	119 [110; 125]	0.004
OS, [IQR]	93 [92; 95]	93 [92; 96]	0.038
Ca/mmol/L, [IQR]	2.37 [2.31; 2.42]	2.42 [2.32; 2.47]	<0.001
LDL/mmol/L, [SD]	2.96 ± 0.52	2.97 ± 0.48	0.630
Smoking Status (%)			0.480
Never	445 (47.3%)	106 (45.1%)	
Quitted	71 (7.6%)	14 (6.0%)	
Smoking	424 (45.1%)	115 (48.9%)	
SCOPAAUT SCORE [IQR]	5 [3; 9]	5 [3; 9]	0.870

SD, mean ± standard deviation; IQR, interquartile range.

### 3.2 Outcome data

This study utilized both univariate and multivariate regression analyses to determine the factors associated with CMVD in high-altitude settings. Univariate analysis was conducted on all variables, and those with *P* < 0.05 (age, sex, SBP, and serum calcium) were subsequently included in the multivariable model. The findings from the unadjusted analysis indicated that age, sex, serum calcium, and SBP were significantly associated with CMVD. These variables were then entered into the multivariable logistic regression model. After adjusting for other variables, the analysis showed that each 1 mmol/L increase in calcium was associated with a 39% higher odds of CMVD (OR = 1.39, 95% CI: 1.20–1.61, *P* < 0.001). Multivariable analysis also identified age, sex, and SBP as independent correlates of CMVD.

In the age-stratified analysis, using <22 years as the reference group, the odds of CMVD were not significantly elevated for the <22 years group (OR = 1.10, 95% CI: 0.78–1.54, *P* = 0.596). For the 22–24 years group, the odds increased significantly (OR = 1.37, 95% CI: 1.03–1.81, *P* = 0.03). Similarly, the 24–27 years group showed a significantly higher odds (OR = 1.36, 95% CI: 1.04–1.78, *P* = 0.026). The highest odds were observed in the >27 years group (OR = 1.75, 95% CI: 1.30–2.35, *P* < 0.001). Overall, the odds of CMVD increased progressively with age beyond 22 years. Additionally, each one-year increase in age was associated with a 5% higher odds of CMVD (OR = 1.05, 95% CI: 1.02–1.09), and each 1 mmHg increase in SBP was associated with a 2% higher odds (OR = 1.02, 95% CI: 1.00–1.03) (Table 2).

**Table 2:** Logistic regression analysis of CMVD on the relationship between CMVD and clinical characteristics after plateau exposure.

Variables	Univariate		Multivariate	
	OR (95% CI)	*P* value	OR (95% CI)	*P* value
Age	1.07 (1.03–1.10)	<0.001	1.05 (1.02–1.09)	0.001
Female	4.11 (1.53–11.06)	0.005	4.13 (1.44–11.89)	0.009
SBP	1.02 (1.01–1.03)	0.003	1.02 (1.00–1.03)	0.009
OS	1.01 (0.98–1.04)	0.55		
Ca	1.39 (1.20–1.60)	<0.001	1.39 (1.20–1.61)	<0.001

CI, confidence interval.

### 3.3 Subgroup analysis

Subgroup analysis explored the relationship between various covariates and CMVD occurrence. Age was segmented into four quartiles: Body Mass Index (BMI) was categorized into four distinct classifications: underweight, normal weight, overweight, and obesity. Other variables were included as continuous values in the logistic regression analysis, as indicated in Figure 2:

Age: With the increase of age, the risk of CMVD gradually increases, especially for people over 27 years old, the risk significantly increases (OR: 1.75, 95% CI: 1.30–2.35, *P* = 0.001).
Gender: Compared with females, males had a lower odds of CMVD (OR = 1.39, 95% CI: 1.20–1.61, *P* < 0.001). The estimate for females showed a wide confidence interval (OR = 1.80, 95% CI: 0.68–4.72, *P* = 0.234), likely due to the small sample size in this subgroup (*n* = 16). These sex-stratified findings should therefore be considered exploratory only and require validation in larger, well-powered cohorts with balanced sex representation.
BMI: In the normal weight (OR: 1.40, 95% CI: 1.18–1.66) and overweight population (OR: 1.41, 95% CI: 1.04–1.92), the risk of CMVD occurrence increased significantly (*P* < 0.05), while the risk variations in the Low body weight and obese groups were not substantial.
Smoking status: In the subgroup analysis, never smokers had an OR of 1.49 (95% CI: 1.20–1.86, *P* < 0.001), current smokers had an OR of 1.26 (95% CI: 1.03–1.54, *P* = 0.027), and former smokers had an OR of 1.61 (95% CI: 0.88–2.92, *P* = 0.119). No significant association was observed for former smokers.

**Figure 2:**
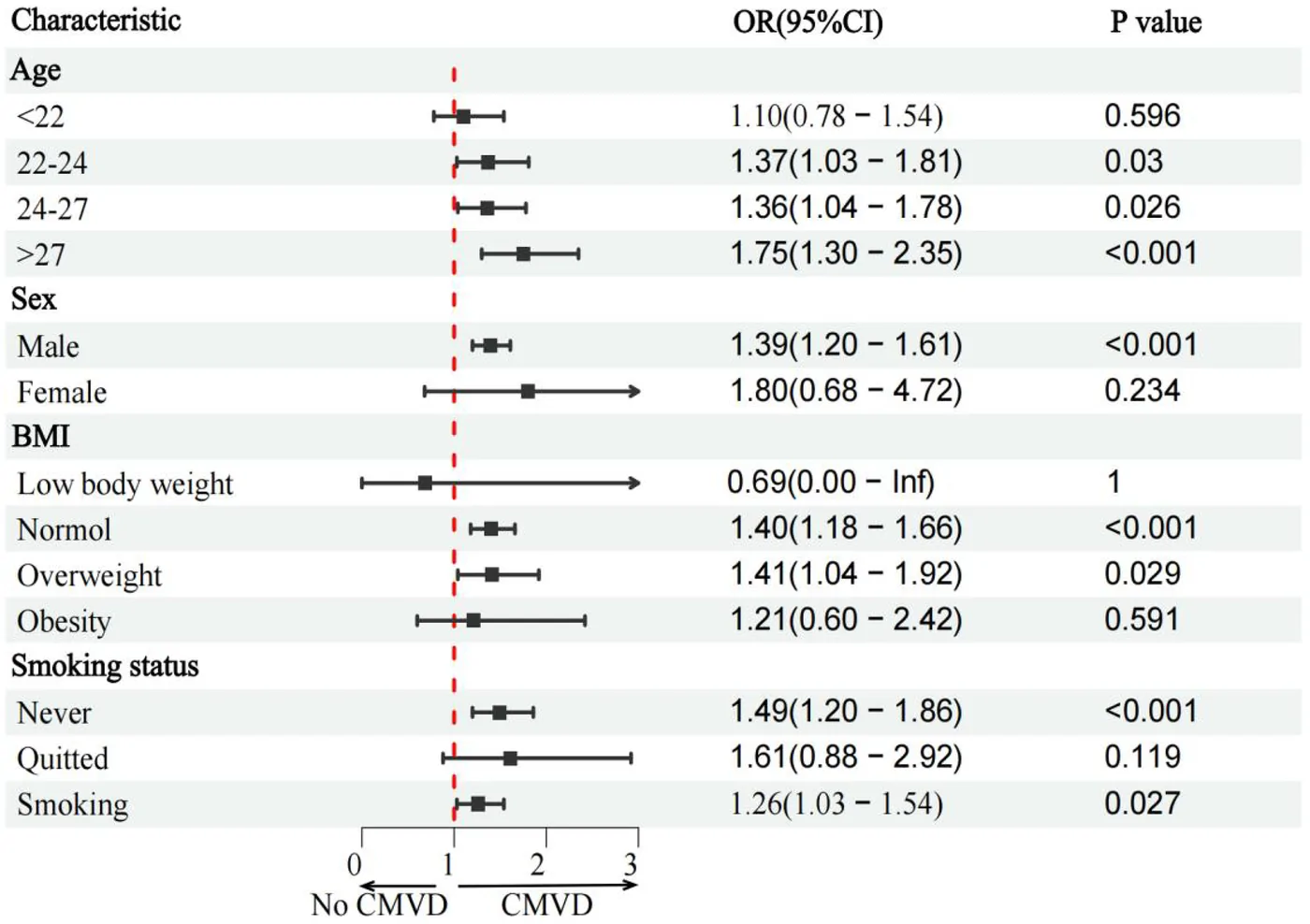
Subgroup analysis of the association between serum calcium and CMVD, stratified by age, sex, BMI category, and smoking status. Note: Subgroup analyses were conducted considering variables such as age, gender, BMI, and smoking status. Age adjustments were made while accounting for BMI, SBP, pre-high-altitude LDL-C, smoking status, and the post-high-altitude SCOPAAUT SCORE. Gender was included in the analysis without any adjustments. For BMI, corrections were applied based on age, SBP, pre-high-altitude LDL-C, smoking status, and post-high-altitude SCOPAAUT SCORE. Additionally, smoking status was adjusted for age, SBP, pre-high-altitude LDL-C, and post-high-altitude SCOPAAUT SCORE.

## 4 Discussion

The objective of this research was to examine the potential association between baseline serum calcium concentrations and CMVD prevalence in high-altitude settings. Based on this nested case-control analysis of a population training at 3,000–3,500 m above sea level, with outcome assessment via initial ECG screening and functional confirmation by transthoracic Doppler echocardiography-derived coronary flow reserve (TDE-CFR), we found that pre-altitude serum calcium level was independently associated with CMVD (each 1 mmol/L increase was associated with a 39% higher odds). Additionally, advanced age and elevated systolic blood pressure were also identified as independent correlates. These findings demonstrate, for the first time in a high-altitude population, a positive association between serum calcium and CMVD, suggesting that serum calcium may have potential value for risk stratification in high-altitude training and stationed populations.

Serum calcium must be maintained within narrow physiological ranges to support cellular function. Observational data indicate that elevated serum calcium levels are associated with an increased risk of coronary artery disease (CAD), with each standard deviation increase in serum calcium corresponding to an 8% rise in the likelihood of cardiovascular events (19). While these findings are derived from CAD populations and are therefore only indirectly suggestive for CMVD, they provide a useful starting point. Several mechanisms may explain this association, including the promotion of vascular calcification, vascular cell dysfunction, enhanced coagulation, and changes in gene expression in the arterial wall mediated by the calcium-sensing receptor (CaSR).

Previous experimental work has demonstrated that calcium plays a critical role in hypoxia-induced vasoconstriction. Voelkel et al. showed that calcium addition augmented the blunted hypoxic pressor response in high-altitude rat lungs, suggesting altered calcium handling in the pulmonary vasculature under chronic hypoxia (20). More recently, Dai and Khalil comprehensively reviewed calcium signaling dynamics in vascular cells, highlighting that dysregulated Ca^2+^ signaling impairs nitric oxide production, reduces vasodilatory capacity, and increases vascular permeability, while in vascular smooth muscle cells it increases vascular tone and promotes phenotypic switching and vascular calcification (21). Additionally, the interplay between calcified atherosclerosis and coronary microvascular dysfunction has been documented in large clinical cohorts, where each 100-unit increase in coronary artery calcium was associated with a 4% higher odds of microvascular dysfunction. However, these studies primarily focused on epicardial atherosclerosis rather than isolated microvascular disease. It is important to emphasize that CAD and CMVD are pathophysiologically distinct: CAD involves large-vessel obstructive atherosclerosis, whereas CMVD is characterized by endothelial dysfunction, smooth muscle hyperreactivity, and microvascular rarefaction. Therefore, evidence from CAD populations should not be directly extrapolated to CMVD, and our findings should be interpreted within the specific context of coronary microvascular pathophysiology.

Among individuals scheduled for training at 3,000–3,500 m, baseline serum calcium levels were found to have an independent and positive correlation with CMVD. Following multivariate adjustment, a rise of 1 mmol/L in Ca^2+^ was associated with a 39% higher odds of CMVD. The observed association is biologically plausible given the known role of calcium in vascular smooth muscle contraction and endothelial function, particularly under hypoxic conditions where calcium homeostasis may be perturbed. Importantly, Salvi et al. demonstrated that high-altitude exposure induces significant microcirculatory modifications, including reduced microcirculatory flow in small blood vessels alongside compensatory increases in capillary density, highlighting the vulnerability of the microcirculation under hypobaric-hypoxic conditions (22).

The odds of CMVD were markedly elevated in females compared to males, with age and systolic blood pressure also recognized as independent correlates. However, it is essential to approach the interpretation of findings related to women with caution, as the small sample size within this subgroup could have resulted in unreliable estimates. These findings suggest that calcium homeostasis may serve as a measurable marker of coronary microcirculatory vulnerability under conditions of hypoxia and sympathetic activation.

In high-altitude environments, potential mechanisms by which calcium may mediate CMVD risk include: (1) Vascular smooth muscle tone and microcirculatory resistance—Increased calcium influx may elevate basal smooth muscle tone and reduce vasodilator reserve, an effect amplified under hypoxia and sympathetic excitation (23); (2) Coagulation and platelet activation—Elevated serum calcium is associated with shortened coagulation time and enhanced thrombotic resilience, potentially worsening microvascular perfusion (24); (3) Endothelial function and vascular calcification phenotype—CaSR and downstream signaling may impair endothelial nitric oxide bioavailability and microvascular reactivity (25). Together, these pathways may predispose individuals to microcirculatory ischemia under high-altitude stress.

The notably higher odds ratio (OR = 1.75) in the >27-year-old group is likely attributable to the convergence of age-related microvascular aging—characterized by increased arterial stiffness and diminished endothelial function—with the cumulative burden of subclinical cardiovascular risk factors, which collectively reduce vasodilator reserve and are synergistically amplified by elevated serum calcium under the stress of high-altitude hypoxia and sympathetic activation, thereby predisposing these individuals to a significantly greater risk of CMVD. This interpretation is consistent with findings from recent large cohorts showing that older age is independently associated with lower myocardial blood flow reserve, and that this association is mediated in part by calcium-related vascular pathways.

In summary, the findings of this research indicate that serum calcium concentrations prior to exposure to high altitudes are significantly associated with CMVD in individuals participating in high-altitude training. Furthermore, these serum calcium levels may serve as a potential screening indicator for CMVD in these specific circumstances. Elevated serum calcium concentrations are recognized correlates of cardiovascular disease and are closely related to various cardiovascular risk factors, including hypertension, dyslipidemia, and diabetes (26–28). However, given that most prior evidence linking calcium to cardiovascular outcomes comes from CAD populations, our findings should be interpreted as preliminary and hypothesis-generating for CMVD specifically. Consistent with this line of investigation, a recent study from our own group using the same high-altitude training cohort demonstrated that higher pre-altitude serum potassium levels were inversely associated with CMVD (OR = 0.26, 95% CI: 0.14–0.47), further supporting the role of electrolyte homeostasis in coronary microvascular health under hypoxic conditions (29). Identifying serum calcium as a potential screening indicator may suggest the potential for targeted screening strategies for high-altitude training populations, such as athletes and military personnel, but prospective validation is needed.

Integrating the monitoring of Ca^2+^ levels into standard pre-training evaluations may facilitate risk identification; however, prospective studies are needed to determine whether this approach improves clinical outcomes.

### 4.1 Limitations and strengths

This study has several methodological strengths, including a relatively large sample size, objectively verified outcomes (ECG screening combined with TDE-CFR confirmation), and the use of a multivariate model to adjust for key confounders. Notwithstanding these strengths, several limitations merit attention.

First, this was a single-center study conducted at the 920th Hospital of the Joint Logistics Support Force, which may limit the generalizability of our findings to other populations or geographic regions with different environmental conditions.

Second, although the overall sample size was substantial, the study population consisted predominantly of healthy males, with only 16 female participants (1.4%). This sex imbalance reflects the actual demographic composition of the military training cohort, where female personnel are substantially fewer than males. The limited number of female participants may have resulted in underpowered sex-specific subgroup analyses, yielding imprecise estimates that require verification in cohorts with a higher proportion of females.

Third, although the nested case-control design with baseline exposure measurement and prospective outcome assessment supports causal inference, the observational nature of the study means that residual confounding from unmeasured variables (e.g., dietary calcium intake, physical activity levels, genetic factors, or environmental stressors) cannot be entirely excluded.

Fourth, we relied on total serum calcium rather than ionized calcium, and routine albumin adjustment was not performed. This may have introduced non-differential misclassification bias, as total calcium levels can be influenced by serum albumin concentrations.

Fifth, although transthoracic Doppler echocardiography (TDE) is a clinically accessible and non-invasive method for assessing coronary flow reserve, it is not the invasive gold standard (which requires catheter-based measurement of CFR and the index of microcirculatory resistance). This may have led to some degree of measurement imprecision.

Finally, the relatively homogeneous study population (young, healthy military trainees) may limit the generalizability of our findings to other populations, such as older adults, civilians, or individuals with pre-existing cardiovascular conditions.

## Data Availability

The raw data supporting the conclusions of this article will be made available by the authors, without undue reservation.
